# Translation of the Morphological Hallmarks of Dyserythropoiesis to Objective Morphometric Parameters by Imaging Flow Cytometry

**DOI:** 10.1111/ijlh.14534

**Published:** 2025-07-26

**Authors:** D. V. Despoina Violidaki, O. A. Olof Axler, L. N. Lars Nilsson, A. P. Anna Porwit, M. E. Mats Ehinger

**Affiliations:** ^1^ Department of Clinical Genetics, Pathology and Molecular Diagnostics Office for Medical Services Lund Sweden; ^2^ Division of Pathology, Department of Clinical Sciences Lund University Lund Sweden; ^3^ Department of Haematology, Oncology and Radiation Physics Skåne University Hospital Lund Sweden

**Keywords:** erythroid dysplasia, erythropoiesis, flow cytometry, imaging flow cytometry, myelodysplastic syndromes

## Abstract

**Introduction:**

Imaging flow cytometry (IFC) is a unique method combining multiparameter flow cytometry (MFC) with morphological evaluation of single cells. Since both analyses are integrated in the diagnostic work‐up of myelodysplastic neoplasms (MDS), we wanted to explore the possibilities of IFC as a diagnostic tool for MDS, with focus on dyserythropoiesis.

**Methods:**

We analysed fresh bone marrow (BM) aspirates from 26 patients with untreated MDS and MDS/MPN and compared them with 12 normal BM specimens (NBM) exploring the cytoplasmic compartment, nuclear abnormalities, and megaloblastoid changes.

**Results:**

The cytoplasmic compartment in MDS showed higher contrast and variance values compared to NBM (*p* < 0.001). Cells with abnormal nuclei and binucleated forms were significantly increased in MDS compared to NBM (*p* < 0.05 for both features). Most binucleated forms were found in the mature compartment, and many of them were G1 phase arrested. All maturation stages showed a significant increase in cell size in MDS compared to NBM (*p* < 0.001). In addition, we found decreased nuclear condensation combined with increased cell size for all erythropoietic maturation stages in MDS compared to NBM (*p* < 0.001). Finally, our previously described MDS‐specific aberrant population of mature erythroblasts with decreased expression of CD36 and/or CD71 showed denser chromatin than both the mature erythropoietic MDS cells without immunophenotypic aberrancies (*p* < 0.001) and NBM (*p* = 0.024).

**Conclusion:**

IFC can detect the major morphological changes associated with dyserythropoiesis in MDS, including the novel findings of increased cytoplasmic texture and bilobated non‐proliferating erythroblasts, allowing for objectivity and standardization.

## 1 Introduction

The diagnosis of myelodysplastic neoplasms (MDS) requires a multi‐step, modular approach including morphological evaluation of blood and bone marrow (BM) smears and BM biopsies, multiparameter flow cytometry (MFC) and genetic tests. Integration of all available laboratory results with the clinical data leads to the diagnosis and final subclassification, according to the WHO and International Consensus Classification of Myeloid Neoplasms and Acute Leukemias (ICC) classification systems [1, 2]. Imaging flow cytometry (IFC) is a unique method combining conventional MFC with morphological evaluation of single cells by brightfield microscopy, providing possibilities to both explore disease biology and standardize morphological criteria for disease classification. There is accumulating data supporting the application of this technique in the field of haematology, resulting in a deeper understanding of normal and disease biology [3, 4, 5, 6, 7, 8], and supporting its potential as a diagnostic tool [9, 10]. However, IFC has not yet become routine in clinical practice, possibly due to its lower throughput compared to conventional flow cytometry and the limited optical resolution of brightfield images as compared to light microscopy. On the other hand, the sophisticated, machine‐based morphometric analysis allows comprehensive and objective studies of cell morphology that would be difficult with traditional cytologic assessment. In addition, IFC offers the unique possibilities to study both the morphology of phenotypically aberrant populations and the immunophenotype of cells with morphologically defined aberrant features. This is particularly relevant for the study of myelodysplastic neoplasms, in which the maturation stages of erythropoiesis are phenotypically well defined [11].

MDS and dyserythropoiesis have been recently explored with IFC by Rosenberg et al. [11]. Their group found increased cell size in MDS compared to normal BM, changes that were more pronounced in neoplasms with high‐risk mutations. They also found higher numbers of binucleated cells in immunophenotypically defined proerythroblasts and mature erythroblasts in MDS compared to normal BM by using a machine‐assisted approach. Although they conducted their studies on cryopreserved mononuclear BM cells isolated with a density gradient medium, they did suggest further investigations on fresh, non‐lysed bone marrow for better representation of the erythroid compartment. Here, we used our previously established non‐lysis MFC protocol [12] on fresh bone marrow cells with a “deviation from normal” approach by comparing BM from MDS patients with normal BM specimens. Our main aim was to search for novel measurable and reproducible morphometric parameters that can be used to define and understand dysplastic erythropoiesis including both cytoplasmic and nuclear abnormalities.

## 2 Materials and Methods

### 2.1 Samples

Fresh BM aspirates from 26 patients with untreated MDS or MDS/MPN (MDS group) collected between the years 2018 and 2020 and classified according to the WHO 4th edition [13] were compared to 12 normal BM specimens (NBM group) (*“Normal Samples”* and Supplementary Table 1, in Supporting Information S7).

### 2.2 Flow Cytometric Analysis

#### 2.2.1 Sample Staining and Acquisition

The applied immunophenotypic panel (CD71‐FITC, CD105‐PE, CD117‐ECD, CD36‐PB, CD45‐KO and the DNA dye DRAQ5) was a modification of our previously described non‐lysis ERY‐panel [12] (“*Sample staining and acquisition”* and Supplementary Table 2, in Supporting Information S7).

**FIGURE 1:**
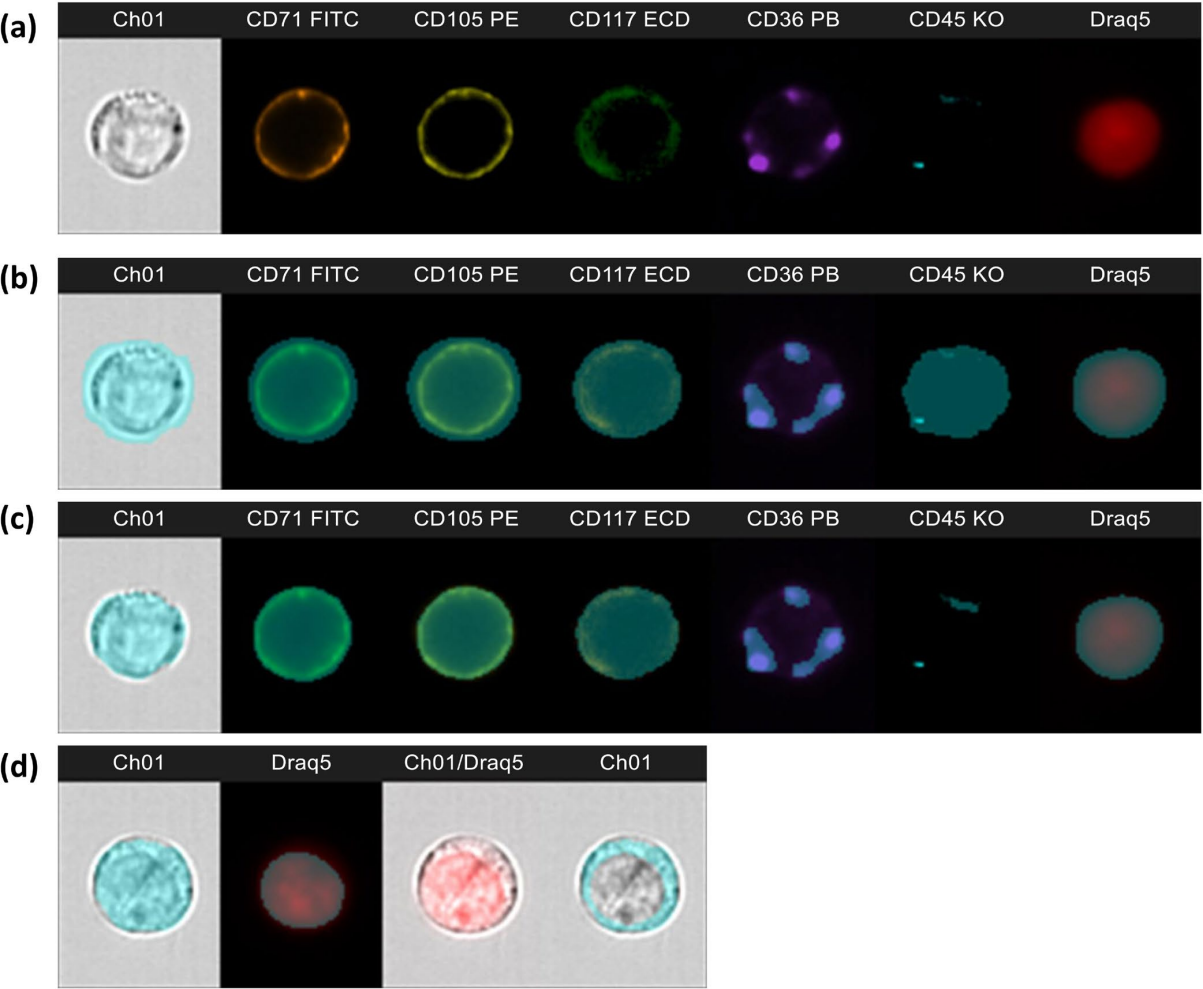
Image gallery of a normal proerythroblast (CD117^+^/CD105^+^) and the applied masks. (a) Brightfield and fluorescence images of the markers used; no masks applied. (b) The default masks provided automatically by the software; note that they often cover larger pixel areas than the ones corresponding to cells or to fluorescence. (c) The same masks after applying the erode function, showing better coverage of the images. (d) From left to right: Cell area defined by eroded mask M01 in the brightfield image; Nuclear area defined by eroded mask M12 in the DRAQ5 channel; Combined cell/nucleus image, no mask applied; Cytoplasmic mask calculated as *Cell Area_*M01 minus *Nuclear Area*_M12.

Acquisition was performed on an Amnis ImageStream^X^ Mk II Imaging flow cytometer (Cytek Biosciences, Fremont, CA, USA), equipped with two cameras (set on 60× magnification) and three lasers (405, 488, and 642 nm), allowing for the acquisition of up to twelve channels. Channels refer to the different detectors that measure the various properties of cells as they pass through the instrument. Channels 1 and 9 provide brightfield images, channel 6 detects side scattered light, and the remaining channels detect fluorescence signals. The channel distribution of the applied panel is presented in Supplementary Table 2, in Supporting Information S7. 50 000 events were collected for each sample, with a live gate set on DRAQ5 positive events. Data analysis was performed in the IDEAS 6.2 software (Amnis/Cytek Biosciences). An example of normal erythroblasts identified by the panel is presented as an IDEAS image gallery in Figure 1a.

#### 2.2.2 Applied IDEAS Masks and Features and Gating Strategies

The IDEAS software provides a wide variety of masks and features for the flow cytometric and morphometric analyses. In essence, a mask is a region of interest, defined as a selection of pixels on an image that differs from the background. Masks can represent brightfield or fluorescence images and are created by default for each channel. Each mask is named according to the corresponding channel, that is, M01 to M12 (Figure 1b). The default masks can then be adjusted by the user or combined to create new ones, in order to more accurately represent the region of interest [14]. The features can be described as mathematical calculations of quantitative and positional data (e.g., intensity or pixel count) performed on the masks. The available features can represent size, shape, location, texture, and signal strength and can be applied to both the brightfield images and the fluorescence signals. The analysed features are provided in Supplementary Table 3 (in Supporting Information S7) and are written in *italics* to visually facilitate the reading of this article. Throughout the text, the feature names are connected with an underscore (_) to a mask name, for example, *Cell Area*_M01, to show to which mask the specific feature was applied. Thus, the cell size is represented by the feature *Area* of the Brightfield mask M01, which from now on will be designated *Cell Area*_M01; the nuclear size is represented by the feature *Area* of the nuclear mask/DRAQ5 M12, designated *Nuclear Area*_M12. Eroded masks M01 and M12 are used (Figure 1c) unless otherwise specified. Finally, the area of the “cytoplasm only” is calculated as *Cell Area_*M01 minus *Nuclear Area*_M12 (Figure 1d).

Optimal cells for assessment: In traditional MFC, gating starts by selecting the live, single events. In IFC, however, a few more gating steps are necessary to retrieve the optimal population for assessment since the morphometric analysis of cell images requires optimally focused cells. The gating strategy we applied was a modification of the one proposed by the IDEAS guided analysis wizard and applied by others (Supporting Information, “*Gating strategy to retrieve the optimal cells for assessment*,” Figure S1) [4, 9, 11, 15].

Erythropoiesis: The erythropoiesis was defined (with the help of Boolean logic) as the CD36^+^/CD45^dim^ OR CD105^+^/CD45^dim^ population [12] (Supporting Information, “*Gating strategy for the Erythroid population*,” Figure S2). To divide erythropoiesis into phenotypically defined maturation stages, corresponding to morphological counterparts (proerythroblasts, basophilic erythroblasts, poly‐ and orthochromatic erythroblasts), we followed the established algorithm [4, 11, 12, 16]: CD117^+^/CD105^+^ ProEry, CD117^−^/CD105^+^ Baso and CD117^−^/CD105^−^ Mature, comprising both poly‐ and orthochromatic erythroblasts (Figure S2c).

**FIGURE 2:**
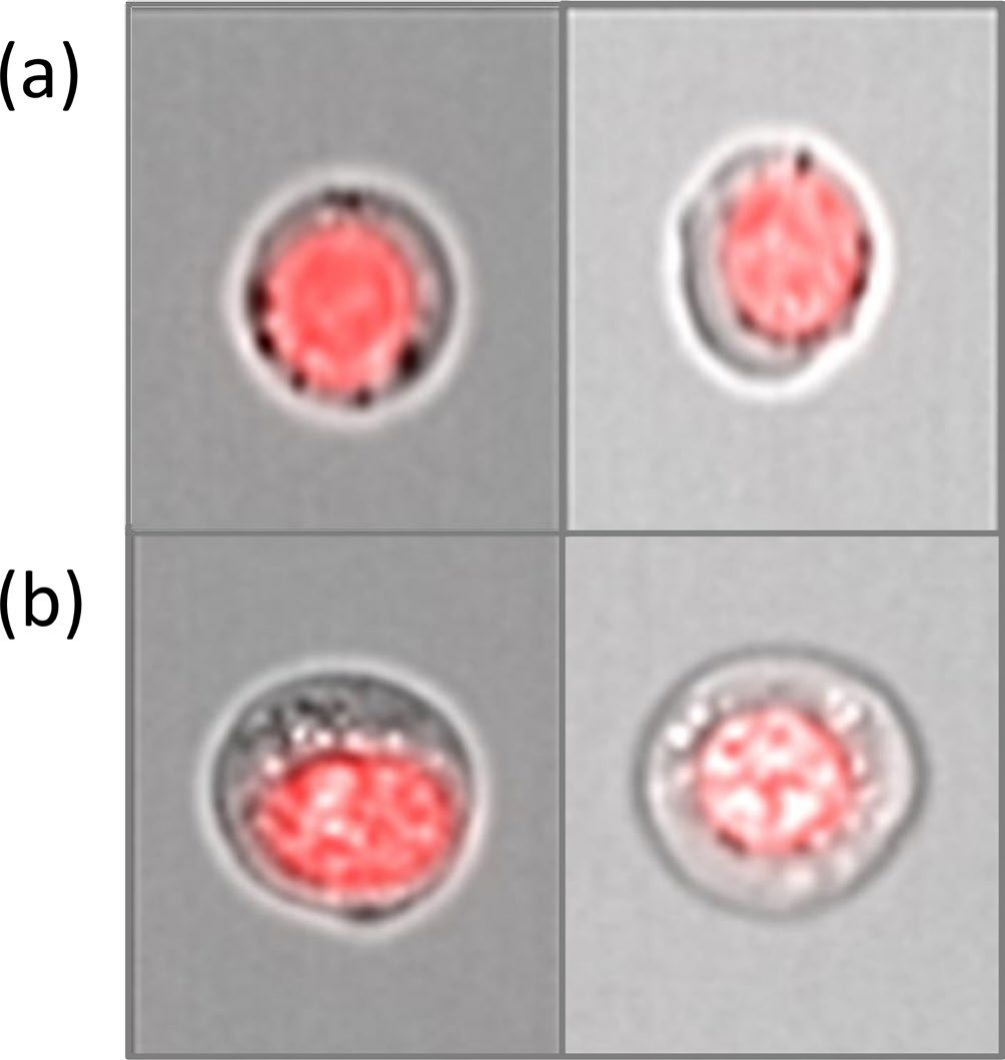
(a) Dark “spots” around the nucleus in a case of MDS with 75% ring sideroblasts. The cells could represent ring sideroblasts. (b) Erythroblasts with clear areas in the cytoplasm that could represent cytoplasmic vacuoles.

Cytoplasmic texture: Under light microscopy, several cytoplasmic changes (such as ring sideroblasts and vacuoles) are characteristic of dyserythropoiesis [13, 17] and could be visualized in the brightfield images (Figure 2). However, these changes could not be quantified with available masks. Thus, instead of defining specific cytoplasmic details, we chose to study the cytoplasmic compartment and its complexity by employing the texture features. The IDEAS software provides several features for the evaluation of texture, including the Haralick texture features [18], of which some (*Contrast*, *Energy*, *Variance*) are used to measure variation, whereas others (*Correlation*, *Entropy*, *Homogeneity*) measure uniformity of the cellular granularity, which is defined as the alternation of darker and brighter areas. A detailed explanation of the texture features used is given in Supplementary Table 3 (in Supporting Information S7).

Nuclear abnormalities: To evaluate the abnormal nuclear shape in general, we used the *Aspect Ratio Intensity* of DRAQ5, which is the Aspect Ratio of the nuclear mask M12, intensity weighted. We used 0.8 as a cut off value, which was calculated as the mean—2SD derived from the NBM group. This value gave good results detecting asymmetrical, abnormal nuclei, confirmed by manual inspection of the derived populations. The detection of binucleated erythroblasts (Supporting Information, “*Gating strategy for the detection of binucleated erythroblasts and nuclear abnormalities”* and Figure S3) was more challenging; it was based on the *Count Lobe* feature which provided a mixture of real binucleates and cells with abnormal nuclei. Therefore, we added a manual step, which included visual confirmation of the real binucleated cells. Of note, we did test the previously applied strategy for the retrieval of binucleated, based on the watershed mask, as described by Rosenberg's group [11], but this method was not reproducible in our hands.

Megaloblastoid changes: The phenomenon of megaloblastoid change is defined as an increase in cell size and asynchronous maturation of the nucleus and cytoplasm [17]. The maturation of the nucleus is characterized by its condensation, a process that could also be described as a specific amount of DNA being “packed” into a decreasing area. To elucidate this phenomenon, we needed a parameter defining nuclear condensation. Hence, we used the feature *Bright Detail Intensity* of DRAQ5 (*BDI‐DRAQ5*), which measures the intensity of brighter areas within a broader masked area (within the nuclear mask M12 corresponding to DRAQ5). The ratio of *BDI‐DRAQ5_*M12‐to‐*Nuclear Area*_M12 could therefore be used as a marker of condensation, where an increasing ratio reflects more nuclear condensation.

### 2.3 Statistical Analyses

Independent‐samples t‐test was applied to assess differences in the mean values of the analyzed parameters between MDS and NBM groups and paired‐samples *t*‐test to assess the difference in the values between different parameters in the same sample/patient. One‐way analyses of variance (ANOVA and MANOVA) were used for comparisons between more than two independent groups. The significance threshold was set at 0.05, with Bonferroni correction applied when multiple hypotheses were tested. The effect size (Cohen's *d*) was calculated whenever statistically significant differences were observed. The values obtained for most tests were > 0.8, suggesting that the observed differences were substantial and likely to be of practical importance. For the sake of simplicity, only Cohen's *d* values below 0.8 were written out in the text along with the significant *p*‐values. The software program used was IBM SPSS statistics 29.0 (Chicago, IL).

## 3 Results

### 3.1 Cytoplasmic Changes

To assess the overall texture of the cytoplasm, we calculated the Haralick texture features [18] *Contrast*, *Correlation*, *Energy*, *Entropy*, *Homogeneity*, and *Variance* (“*Texture features*” and Supplementary Table 3, in Supporting Information S7) for the cytoplasmic mask, which was created by subtracting the nuclear mask M12 from the cell mask M01 (Figure 1d). We compared these features between MDS and NBM groups for the total of erythropoiesis, and we observed higher mean and median values in MDS compared to NBM for the features *Contrast* and *Variance* (*p* < 0.001 for both features, Figure S4). We repeated the test for lymphocytes as a negative control (low anticipated cytoplasmic texture) and for the granulocytes as a positive control (richer texture due to granules). As expected, we observed lower values for the *Contrast* and *Variance* features in lymphocytes and higher values for the granulocytes. Neither of these two populations showed any difference between the MDS and NBM groups for any of the texture features (Figure S4).

To explain the finding of higher texture values in erythropoiesis in MDS compared to NBM, we considered the possibility that the increased texture could be due to an increased presence of siderotic granules including ring sideroblasts. We therefore compared the texture features of the MDS and MDS/MPN cases with > 15% ring sideroblasts (*n* = 10 of 26) with the rest of the MDS cases lacking ring sideroblasts (*n* = 16 of 26). Interestingly, there was no significant difference in any of the Haralick texture features (data not shown), suggesting that cytoplasmic content other than siderotic granules could be responsible for the increased cytoplasmic texture in MDS samples.

### 3.2 Nuclear Abnormalities

**FIGURE 3:**
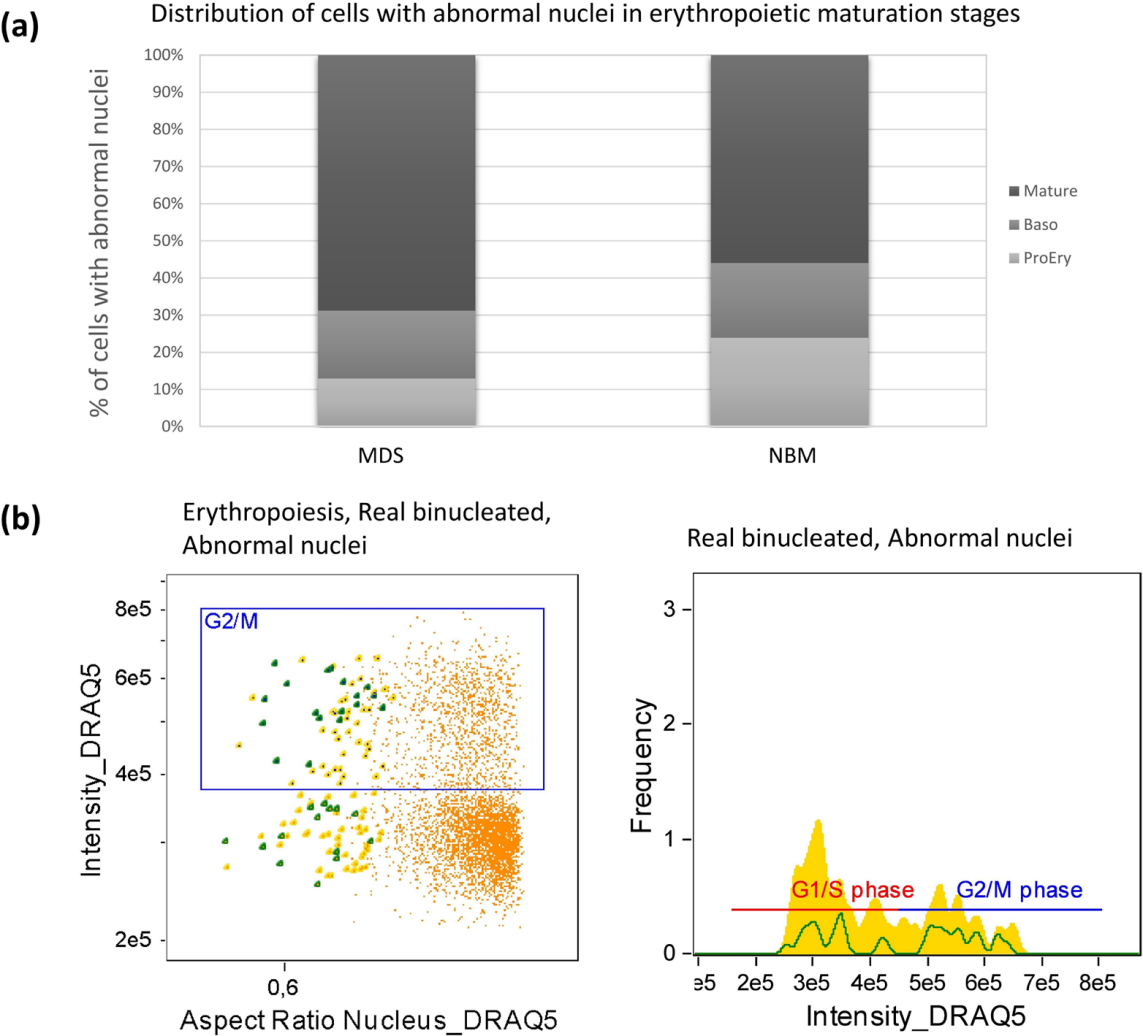
(a) Distribution of cells with abnormal nuclei in the three maturation stages, in MDS and normal BM cases. In the MDS cases, most cells (66.6%) with abnormal nuclei were mature whereas 18.3% and 15.1% of them were found in the Baso and ProEry compartments, respectively. In NBM there was a similar distribution of abnormal nuclei between mature (56%) and early stages (44%) distributed also equally between ProEry (22.8%) and Baso (21.2%). (b) Scatter plot of Aspect ratio of the nuclear mask M12 versus DRAQ5 intensity (left) and histogram of DRAQ5 intensity (right) of a representative MDS case. In both diagrams the G2/M gate highlights the proliferating cells (erythropoiesis—orange, abnormal nuclei—yellow, real binucleated—dark green). Some of the real binucleated were in the G1 phase suggestive of non‐dividing cells, whereas others exhibited twice the DRAQ5 intensity, consistent with proliferation.

Cells with abnormal nuclei, excluding binucleates, were found more often in the MDS cases constituting on average 11.5% of total erythropoiesis (median 10.9%, range 5.3%–19.9%) versus 9.1% (median 9.2%, range 6.1%–12.9%) in the NBM cases (*p* = 0.017, Cohen's *d* = 0.73) (Table S4). In the MDS cases, most cells with abnormal nuclei were mature (66.6% in MDS vs. 54% in NBM, *p* = 0.001, Figure 3a). However, in the early stages (ProEry and Baso) there was no difference regarding the number of cells with abnormal nuclei between MDS and NBM. Taking a closer look at binucleated cells alone, we found on average 22.4 of them (range 4–51, mean 0.9% and median 0.7% of total erythropoiesis) in the MDS cases and 7.8 cells (range 1–17, mean and median 0.4% of total erythropoiesis) in the NBM group (*p* < 0.001) (Supplementary Table 4, in Supporting Information S7). The distribution in maturation stages was similar as with the cells with abnormal nuclei, with a significantly higher number of binucleated cells observed in the mature compartment in MDS compared to NBM (73% in MDS vs. 57% in NBM, *p* < 0.001), whereas there was no difference observed in the earlier stages (ProEry and Baso together, 27% in MDS vs. 43% in NBM) (Figure S5). Interestingly, analysis of the DRAQ5 content in the cells with abnormal nuclei and the binucleated cells showed two subpopulations: one with an intensity consistent with euploid DNA status, representing the G1 phase of the cell cycle, and one with twice the DRAQ5 intensity of the euploid cells, consistent with the G2/M phase of the cell cycle and thus proliferation (Figure 3b). These euploid binucleated cells were found significantly more often (*p* = 0.014) in MDS cases (average 58.4%, range 26.3%–92.8%) by comparison to NBM (average 34.8%, range 0%–82.3%). By contrast, the proliferating binucleated forms were found in higher numbers in the normal compared to the MDS cases (mean 41.6% in MDS vs. 65.2% in NBM, *p* = 0.013, Cohen's *d* = 0.60).

### 3.3 Megaloblastoid Changes

**FIGURE 4:**
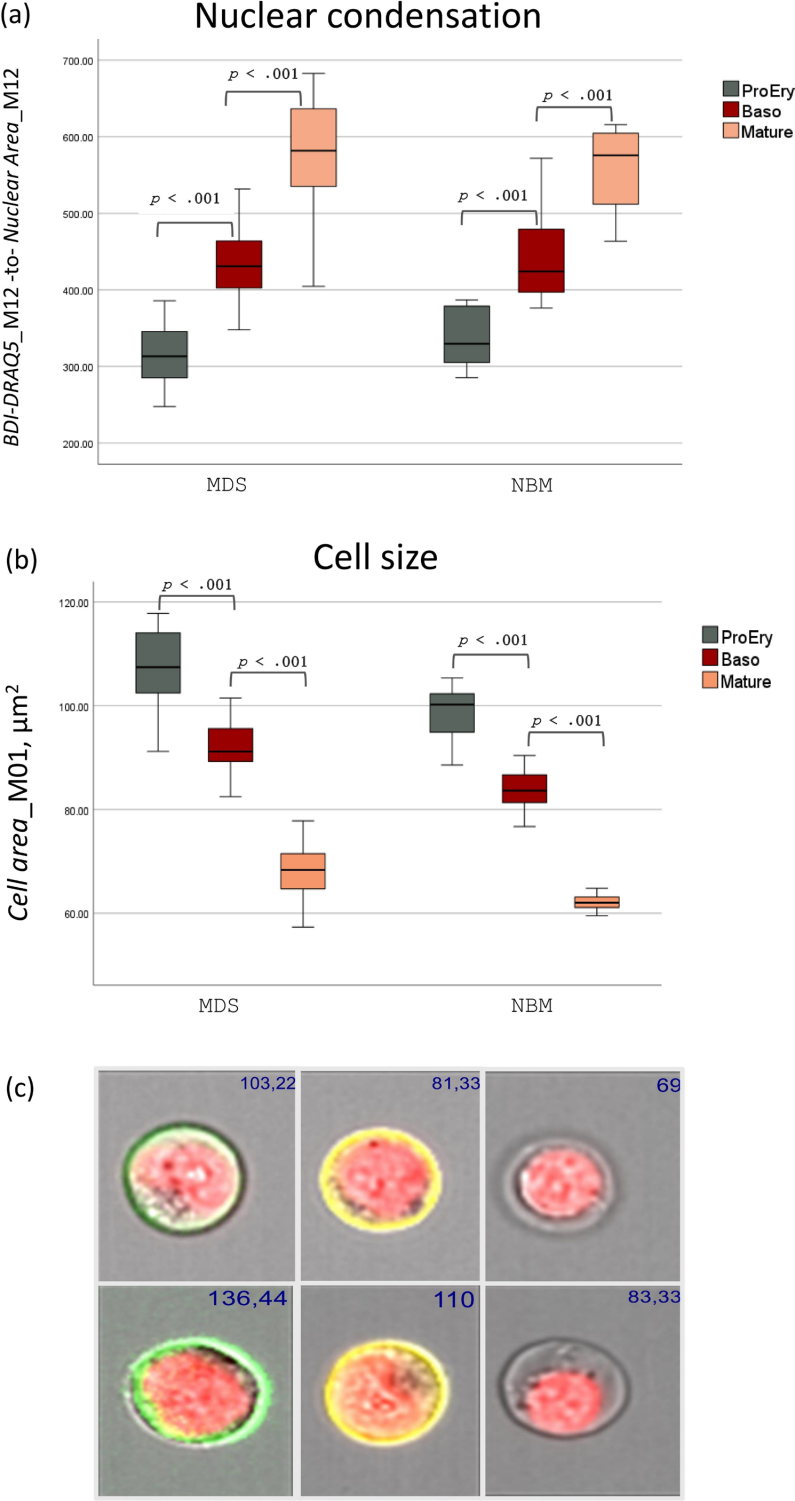
(a) Boxplot depicting changes in nuclear condensation (measured as the ratio *BDI‐DRAQ5_*M12—to—*Nuclear Area*_M12) in the three phenotypical maturation stages in MDS patients (MDS, left) and normal controls (NBM, right). (b) Boxplot depicting cell size changes in the three phenotypical maturation stages in MDS patients (MDS, left) and normal controls (NBM, right). (c) Image gallery showing the sequential decrease in cell size from a proerythroblast (CD117^+^, green), basophilic erythroblast (CD105+, yellow) to a mature erythroblast. Upper panel: Normal case. Lower panel: MDS case with prominent megaloblastoid changes. The values in blue font in the right upper corner represent the cell area (μm^2^).

We used the IDEAS software feature *Bright Detail Intensity* of DRAQ5 for assessment of nuclear condensation to further study the phenomenon of megaloblastoid change. As expected, we observed increased nuclear condensation following maturation when we compared the maturation stages, both within the MDS and the NBM groups (*p* < 0.001 for all comparisons between maturation stages, Figure 4a,c and Table 1). The differences in nuclear condensation between MDC and NBM were not statistically significant, neither for the total erythropoiesis nor for each maturation stage separately (Table 1). However, when we specifically studied enlarged cells, defined as the mean + 2SD of the cell size of the normal erythroblasts of each corresponding stage [11], we observed statistically significant (*p* < 0.001) lower nuclear condensation in all maturation stages of MDS erythropoiesis compared to the corresponding maturation stages of the NBM group (Table 1). We could also confirm the previously described [11] increase in the mean cell size of the total erythropoiesis and of each of the three phenotypically described maturation stages (ProEry, Baso, Mature) in the MDS patients compared to the NBM (*p* < 0.001 for all comparisons, Figure 4b,c and Supplementary Table 5 in Supporting Information S7). Regarding the nuclear: cytoplasm ratio, we observed a tendency to lower values in MDS compared to the NBM group in all maturation stages, but the difference was statistically significant only for the mature compartment (*p* = 0.02, Cohen's *d* = 0.53).

**TABLE 1:** Values of *BDI‐DRAQ5*_M12‐to‐*Nuclear Area*_M12 (“nuclear condensation”) in NBM and MDS (presented as mean ± SD).

	Total erythropoiesis	ProEry	Baso	Mature
NBM	537 ± 50.8	355 ± 88.6	446 ± 62	556 ± 52
MDS Total populations^a^	554 ± 65.6	321 ± 54	436 ± 57.3	573 ± 70
MDS Only megaloblasts^b^		252 ± 47	347 ± 49	494 ± 64
*p*‐value NBM versus MDS—total^a^	0.45	0.14	0.61	0.45
*p*‐value NBM versus MDS—megaloblasts^b^		< 0.001	< 0.001	< 0.001

^a^ Megaloblasts included.

^b^ Defined as erythroblasts with enlarged size (> mean + 2SD of the cell size of the normal erythroblasts of the same maturation stage).

We next focused on our previously described [12] aberrant population of erythroblasts showing decreased expression of CD36 and/or CD71 (CD36^−/dim^ ± CD71^−/dim^). This population was observed in 23 of 26 (88%) MDS cases and 3 of 12 (23%) NBM (mean 0.3% of erythropoiesis in NBM vs. 6.2% in MDS, *p* < 0.001). As this population belongs to the mature compartment of erythropoiesis (CD117^−^/CD105^−^), we compared it both to the mature population of normal BM and to the mature, non‐aberrant compartment of the MDS cases, from which this population was excluded by using Boolean Logic (Mature AND NOT CD36^−/dim^/CD71^−/dim^). The aberrant CD36^−/dim^ ± CD71^−/dim^ population showed significantly increased cell size compared to the mature compartment of the normal BM (*p* = 0.039, Cohen's *d* = 0.59), but significantly decreased size compared to the mature, non‐aberrant compartment of the MDS cases (*p* = 0.019, Cohen's *d* = 0.71) (Figure S6). The nucleus, on the other hand, was more condensed compared to both the mature erythroblasts of normal BM (*p* = 0.024) and the mature, non‐aberrant MDS population (*p* < 0.001) (Figure S6).

## 4 Discussion

Application of IFC allowed us to discover new morphometric features of dyserythropoiesis while recapitulating some of the traditional morphological findings of abnormal erythroid maturation. A strength of the morphometric analyses provided by IFC is that they ensure objectivity and standardization, characteristics missing from the traditional morphological assessment by light microscopy, in which the interobserver variability is a commonly described limitation [17]. The IDEAS software provides in addition the possibility to incorporate machine‐learning assisted applications [19, 20, 21, 22] which can facilitate the automated, unsupervised analysis and minimize the human factor, thus augmenting objectivity and reproducibility.

In contrast to previous studies of IFC on erythropoiesis [11] we used fresh BM specimens treated with a non‐lysis protocol, ensuring that the whole erythropoietic compartment could be studied unaffected by a freeze‐thawing process. Although cryopreservation has obvious advantages for research, it has been known for a long time that thawed cells are not fully equivalent to fresh bone marrow cells [23]. We consider avoiding lysis imperative, especially for the study of the more mature erythroid compartment, which is the one affected more by lysis [12, 24, 25, 26], and at the same time, as our results show, the one that more often exhibits signs of dyserythropoiesis. The drawback of this approach is the longer acquisition time to reach the required number of nucleated cells.

This is the first study to explore cytoplasmic changes in erythropoiesis by IFC. Although some of the morphologically characteristic cytoplasmic changes, such as ring sideroblasts and vacuoles, could be visualized by IFC (Figure 2), they could not be reliably investigated with morphometric IFC parameters. Instead, we focused on the whole cytoplasmic compartment by employing the texture features. We found significantly higher values for the texture parameters *Contrast* and *Variance* in MDS compared to NBM. Both features are measures of intensity variation in a studied area, indicating more cytoplasmic complexity in MDS compared to normal BM. This finding could not be readily explained in our hands by an increased iron content in the cytoplasm, as there was no significant texture difference between MDS cases with and without high numbers of ring sideroblasts. This suggests that other intracytoplasmic components may contribute to the cytoplasmic complexity, such as mitochondria, endosomes, or other intracytoplasmic vesicles. Interestingly, studies of normal erythropoiesis have shown increased clearance of mitochondria along with an increased number of endosomes, multivesicular bodies, and autophagosomes during maturation [27]. This clearance has been proven essential for erythroid maturation and enucleation [28, 29]. In MDS, both a higher number of mitochondria and impaired mitochondrial gene expression appear to be a feature [30]. Taken together, these findings introduce the possibility that abnormal accumulation of mitochondria in the cytoplasm may contribute to the dysfunctional erythropoiesis characterizing MDS. Further studies will be needed to determine if this is indeed the case. Except for the formation of ring sideroblasts, vacuolization, and aberrant periodic acid–Schiff (PAS) positivity, the quality of the cytoplasm is unrecognized as a defining feature of MDS‐related dysplastic erythropoiesis in current hematopathology practice [1, 2]. Therefore, the results of the present study suggest that assessment of the cytoplasmic compartment by IFC can be used for objective evaluation of dyserythropoietic changes.

IFC also provided a deeper understanding of nuclear abnormalities defining dyserythropoiesis that are used in the daily diagnostic work‐up. We found an increased number of binucleated erythroblasts in MDS cases compared to the normal BM, as previously reported by Rosenberg et al. [11]. Interestingly, by examining these cells in greater detail, we discovered that the binucleated erythroblasts in MDS were often non‐proliferating and G1 phase arrested, as judged by their euploid DNA content. By contrast, binucleated cells in normal BM were more often immature and proliferating, as indicated by their increased staining with DRAQ5, consistent with the G2/M phase of the cell cycle. Hence, euploid binucleated erythroblasts appear to comprise a genuinely abnormal population reflecting abnormal maturation rather than proliferation. As the term binucleated (i.e., with two nuclei) presumes polyploidy, we consider that the euploid “binucleated” cells would in fact be better designated as “bilobated.”

We next focused on nuclear abnormalities other than binucleation. Here we managed to develop a new method to quantify nuclear irregularities by IFC. With our strategy, we found significantly more cells with nuclear abnormalities other than binucleates in the MDS cases, in contrast to Rosenberg's study. The increased frequency of cells with abnormal nuclei and of binucleate cells in MDS was restricted to the mature compartment only, that is, the polychromatic and orthochromatic erythroblasts. Rosenberg et al. found significantly more binucleates in the ProEry compartment as well [11]. However, many of these binucleated cells could in fact be proliferating cells unrelated to G1‐phase arrested bilobated cells, thus providing a possible explanation for the discrepant results between our study and theirs.

Although increased cell size of erythroblasts is a characteristic feature of MDS for all maturation stages (i.e., proerythroblasts, basophilic erythroblasts and mature erythroblasts) as shown in this study and by others [11], it is indeed quite unspecific. For example, increased cell size is typical of megaloblastic anemia and can be found in some congenital anemias. We therefore sought to explore the phenomenon of megaloblastoid change, defined by conventional morphology as asynchronous maturation of the nucleus and cytoplasm in addition to an increase in cell size [17], which has not been studied by IFC before. Since nuclear maturation is morphologically appreciated as denser chromatin, we provided the novel parameter of nuclear condensation, measured as the ratio of *Bright Detail Intensity* of DRAQ5‐to‐*Nuclear Area*. In MDS cases, erythroblasts with increased cell size (defined as the mean + 2SD of the cell size of the normal erythroblasts of each corresponding stage) exhibited in all stages significantly less condensed nucleus, or finer chromatin, compared to NBM. Thus, we managed to define the megaloblast by quantifying the nuclear condensation with IFC, a parameter that previously only subjectively could be assessed by conventional morphology under the light microscope. With respect to the traditional term “asynchronous maturation” our results clearly show that the maturation asynchrony is restricted to enlarged erythroblasts and not ordinary‐sized erythroblasts, that is, normoblasts.

The aberrant mature CD36^−/dim^ ± CD71^−/dim^ population that was significantly expanded in MDS cases showed the highest degree of nuclear condensation, a finding supporting that this compartment represents an abnormal population of cells at a phenotypical maturation state closer to reticulocytes, in which the nucleus should have normally been extruded, but instead is retained heavily condensed [12].

Taken together, our results highlight the objectivity of observations (such as cytoplasmic and megaloblastoid changes) as well as the deeper understanding of disease biology (e.g., the finding of abnormal, G1 phase arrested binucleated erythroblasts) as the main benefits of IFC. At the same time, analysis by IFC entails some degree of complexity and a relatively long learning curve to master the brightfield imagery and the large battery of available features. The unique imaging function of IFC is not equal to light microscopy because brightfield images do not provide information in the same manner as Romanowsky‐stained smears. Still, IFC provides alternative tools for the morphological assessment of cells, with its feature‐based morphometric analyses. That said, the large number of available features and the countless possibilities to combine features and masks can make the design of an analysis protocol time‐consuming. These limitations reflect the need for further studies, including methodological ones, to support the role of IFC, currently applied only in research, as a diagnostic tool. In this context, the application of artificial intelligence and machine learning strategies to interpret IFC data are promising to overcome the current technical limitations of IFC, increase the information yield, and refine diagnostic objectivity [19, 20, 21, 31].

In conclusion, we discovered that increased cytoplasmic complexity, as defined by IFC, is an inherent feature of MDS, thus overcoming the limitations of traditional light microscopy. We also found that many of the binucleated erythroblasts characteristic of MDS are in fact euploid and thus non‐proliferating. Finally, we were able to define megaloblastoid change in a novel, objective manner. Importantly, some of our findings, including the increased frequency of abnormally nucleated or binucleated cells in MDS and the decreased nuclear: cytoplasmic ratio, were restricted to the mature compartment of erythropoiesis, which underscores the benefits of our non‐lysis protocol on fresh bone marrow cells, leaving the whole erythropoietic compartment intact for exploration of dyserythropoietic features. In this study we compared various MDS‐diagnoses with normal BM, but studies with larger number of MDS cases also including non‐clonal cytopenias, which was beyond the scope of this study, could more strongly support the role of IFC in the diagnostic work‐up of MDS. By using a “deviation from normal” approach, we were able to translate the morphological changes of dyserythropoiesis into measurable and reproducible morphometric parameters, setting the ground for further research on MDS by IFC.

## Author Contributions

Conceptualization: Despoina Violidaki, Olof Axler, Lars Nilsson, Anna Porwit, and Mats Ehinger. Methodology: Olof Axler. Validation: Olof Axler. Formal Analysis: Despoina Violidaki. Investigation: Olof Axler and Despoina Violidaki. Resources: Lars Nilsson. Data Curation: Despoina Violidaki. Writing – Original Draft Preparation: Despoina Violidaki. Writing – Review and Editing: Despoina Violidaki, Olof Axler, Lars Nilsson, Anna Porwit, and Mats Ehinger; Visualization: Despoina Violidaki. Supervision: Mats Ehinger. Funding Acquisition: Mats Ehinger and Anna Porwit. All authors have read and agreed to the published version of the manuscript.

## Ethics Statement

The study was conducted according to the guidelines of the Declaration of Helsinki and approved by the Regional Ethical Review Board of Lund, Sweden (Nr 223/2017).

## Conflicts of Interest

The authors declare no conflicts of interest.

## Supporting information


**Figure S1.** Gating strategy to retrieve the optimal cells for assessment. (a) Histogram of *Gradient RMS* of Brightfield (Mask M01) to retrieve the optimally focused cells. (b) The focused cells are subsequently plotted in a scatter plot *Cell Area*_M01 versus *Aspect Ratio*_M01 to retrieve single cells. (c) The single cells are plotted in a histogram of DRAQ5 Intensity to remove the apoptotic cells (low DRAQ5). (d) Scatterplot of the features *Centroid X*_M01‐erode versus *Circularity*_M01‐erode to remove cropped or abnormally shaped cells. (e) Examples of artefacts; from top to bottom: cropped cells, particles, and doublets.

**Figure S2.** Gating strategy for the Erythroid population. (a) Scatter plot of CD45 versus CD36 to retrieve the CD36^+^/CD45^−^ erythroid population. (b) Scatter plot of CD45 versus CD105 to retrieve the earliest CD105^+^/CD45^dim^ erythroid cluster. (c) Total Erythropoiesis (CD36^+^/CD45^−^ AND CD105^+^/CD45^dim^) is subsequently divided into three maturation stages, CD117^+^/CD105^+^ ProEry (black), CD117^−^/CD105^+^ Baso (burgundy) and CD117^−^/CD105^−^ Mature (orange). (d) Approximate gating of other cell lines (granulopoiesis, monocytes, lymphocytes) in a CD45 versus CD36 scatter plot.

**Figure S3.** Gating strategy for the detection of binucleated erythroblasts in a MDS case. (a) Histogram of the *Lobe Count* feature, detecting cells with 2, 3, and 4 lobes. The initial “Bilobed” population in lime green. (b) To determine reference gates for the detection of binucleated cells in the initial “Bilobed” population, real binucleated cells (two examples are shown in the image gallery) were hand‐picked by visual inspection and subsequently displayed (dark green) in the scatter plots *Symmetry 2*_M12 versus *Circularity*_M12 and then *Aspect Ratio intensity*_M12 versus *Compactness*_M12. (c) Next, the initial “Bilobed” population (428 events) was displayed in the same scatter plots as in (b). Note that the “Bilobed” population included both binuclear cells and cells with abnormal nuclear shape (three examples are shown in the image gallery). By applying the reference gates as determined in (b) we ended up in 81 presumably binucleated events (including the 33 real binucleates).

**Figure S4.** Boxplots depicting (a) cytoplasmic *Contrast* and (b) cytoplasmic *Variance* in erythropoiesis (blue), granulocytes (red) and lymphocytes (green) in MDS (left boxes) and NBM (right boxes). Higher *Contrast* and Variance is shown in erythropoiesis in MDS compared to NBM. As expected, no difference is observed in granulocytes and lymphocytes between MDS and NBM.

**Figure S5.** Distribution of binucleated cells in the three maturation stages, in MDS and normal BM cases. In the MDS cases, most binucleated cells (73%) were mature. In NBM there was a similar distribution of abnormal nuclei between mature (57%) and early stages (43%).

**Figure S6.** Boxplots depicting changes in (a) cell size and (b) nuclear condensation (measured as the ratio *BDI‐DRAQ5_*M12 ‐to‐ *Nuclear Area*_M12) in the aberrant CD36^−/dim^ ± CD71^−/dim^ population (blue, left box), in the mature, non‐aberrant compartment of the MDS (red, middle box) and in the mature compartment of NBM (green, right box).

Data S1.

## Data Availability

The data that support the findings of this study are available from the corresponding author upon reasonable request.
