# Supplementary figures and images for: Translation of the Morphological Hallmarks of Dyserythropoiesis to Objective Morphometric Parameters by Imaging Flow Cytometry
Source: Int J Lab Hematol. 2025 Jul 26;47(6):1089–98. doi: 10.1111/ijlh.14534 (PMC12597861; doi:10.1111/ijlh.14534)

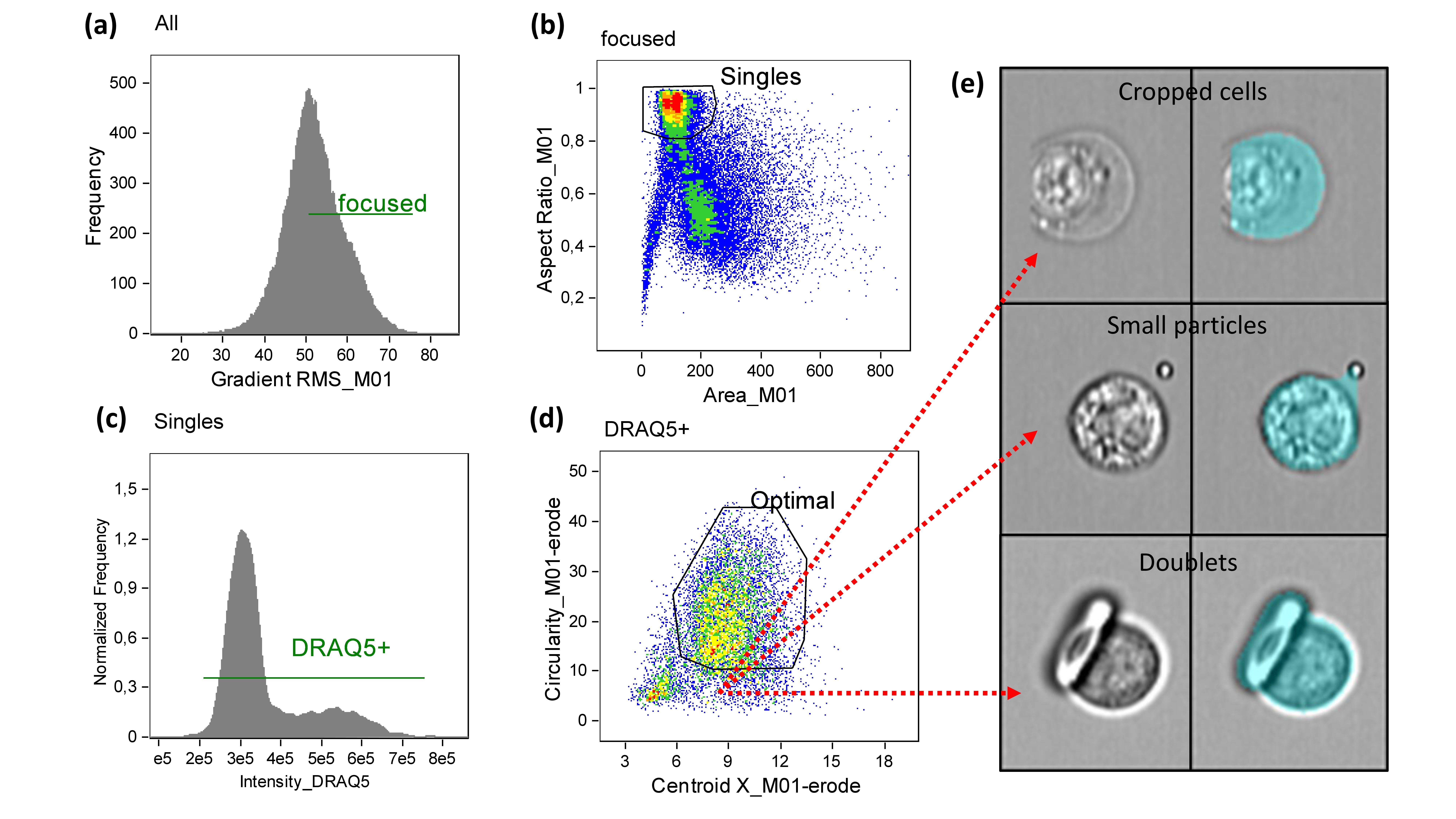

Supplement: Supplementary file 1 — Figure S1. Gating strategy to retrieve the optimal cells for assessment. (a) Histogram of Gradient RMS of Brightfield (Mask M01) to retrieve the optimally focused cells. (b) The focused cells are subsequently plotted in a scatter plot Cell Area_M01 versus Aspect Ratio_M01 to retrieve single cells. (c) The single cells are plotted in a histogram of DRAQ5 Intensity to remove the apoptotic cells (low DRAQ5). (d) Scatterplot of the features Centroid X_M01‐erode versus Circularity_M01‐erode to remove cropped or abnormally shaped cells. (e) Examples of artefacts; from top to bottom: cropped cells, particles, and doublets. [file IJLH-47-1089-s005.jpg]

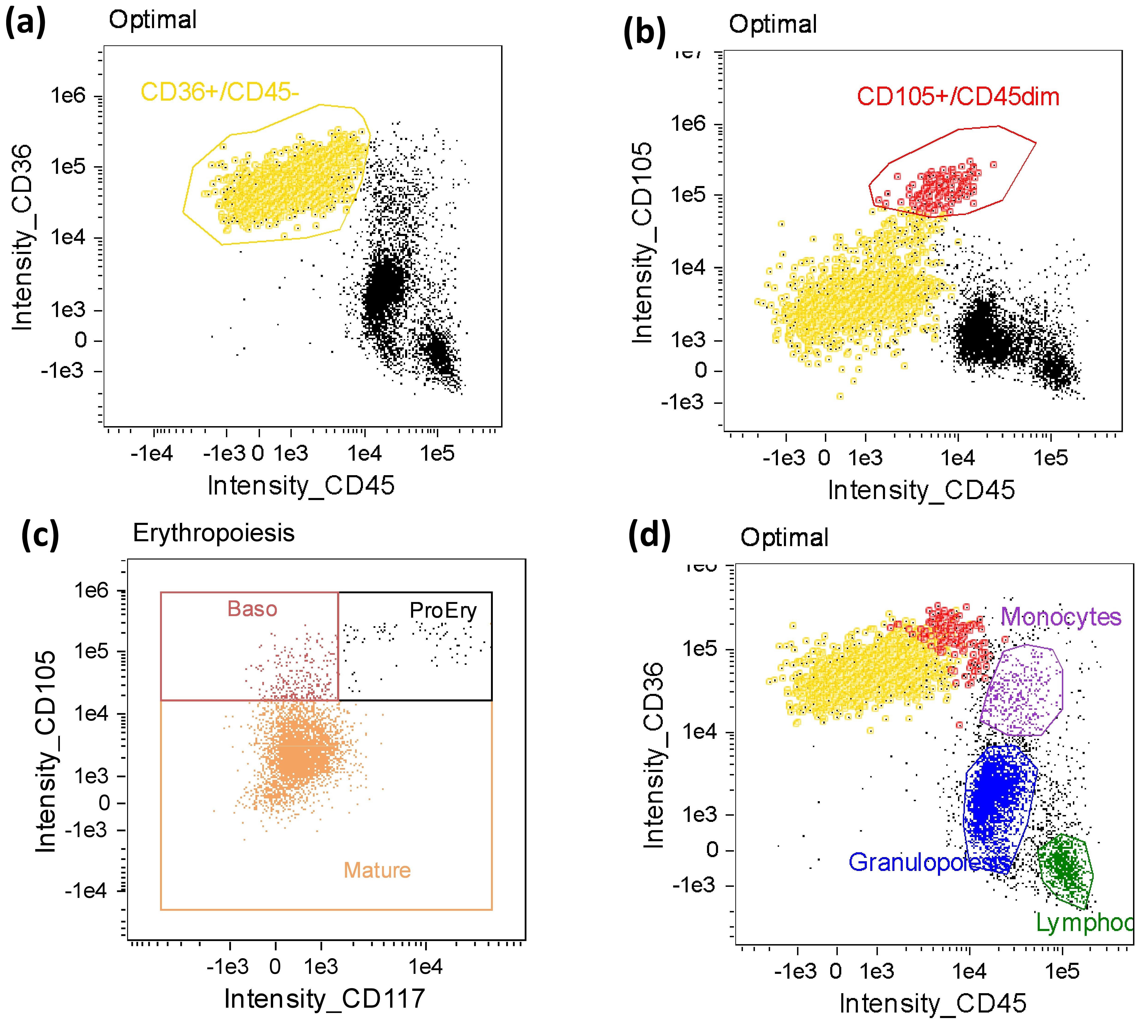

Supplement: Supplementary file 2 — Figure S2. Gating strategy for the Erythroid population. (a) Scatter plot of CD45 versus CD36 to retrieve the CD36+/CD45− erythroid population. (b) Scatter plot of CD45 versus CD105 to retrieve the earliest CD105+/CD45dim erythroid cluster. (c) Total Erythropoiesis (CD36+/CD45− AND CD105+/CD45dim) is subsequently divided into three maturation stages, CD117+/CD105+ ProEry (black), CD117−/CD105+ Baso (burgundy) and CD117−/CD105− Mature (orange). (d) Approximate gating of other cell lines (granulopoiesis, monocytes, lymphocytes) in a CD45 versus CD36 scatter plot. [file IJLH-47-1089-s006.jpg]

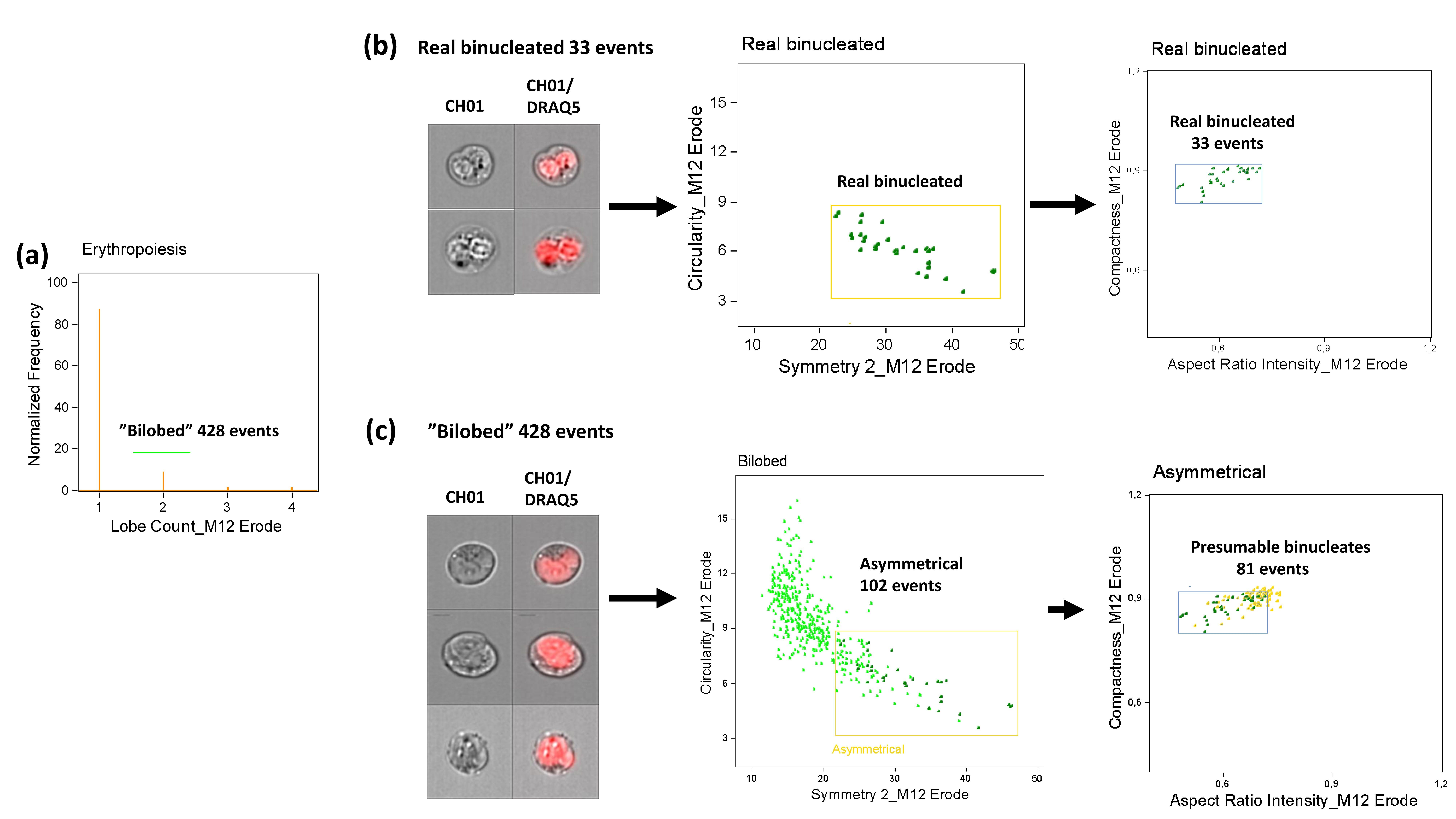

Supplement: Supplementary file 3 — Figure S3. Gating strategy for the detection of binucleated erythroblasts in a MDS case. (a) Histogram of the Lobe Count feature, detecting cells with 2, 3, and 4 lobes. The initial “Bilobed” population in lime green. (b) To determine reference gates for the detection of binucleated cells in the initial “Bilobed” population, real binucleated cells (two examples are shown in the image gallery) were hand‐picked by visual inspection and subsequently displayed (dark green) in the scatter plots Symmetry 2_M12 versus Circularity_M12 and then Aspect Ratio intensity_M12 versus Compactness_M12. (c) Next, the initial “Bilobed” population (428 events) was displayed in the same scatter plots as in (b). Note that the “Bilobed” population included both binuclear cells and cells with abnormal nuclear shape (three examples are shown in the image gallery). By applying the reference gates as determined in (b) we ended up in 81 presumably binucleated events (including the 33 real binucleates). [file IJLH-47-1089-s003.jpg]

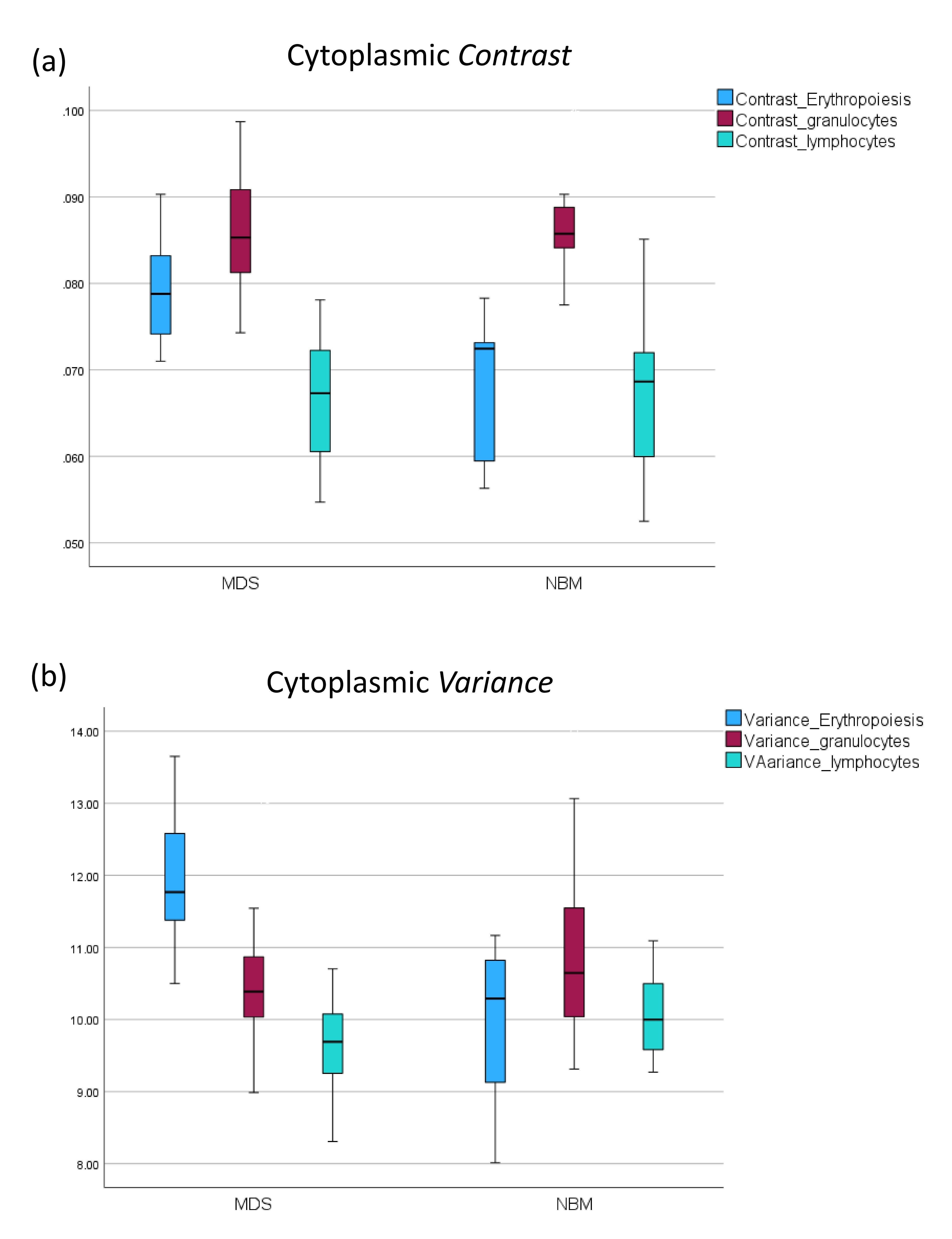

Supplement: Supplementary file 4 — Figure S4. Boxplots depicting (a) cytoplasmic Contrast and (b) cytoplasmic Variance in erythropoiesis (blue), granulocytes (red) and lymphocytes (green) in MDS (left boxes) and NBM (right boxes). Higher Contrast and Variance is shown in erythropoiesis in MDS compared to NBM. As expected, no difference is observed in granulocytes and lymphocytes between MDS and NBM. [file IJLH-47-1089-s004.jpg]

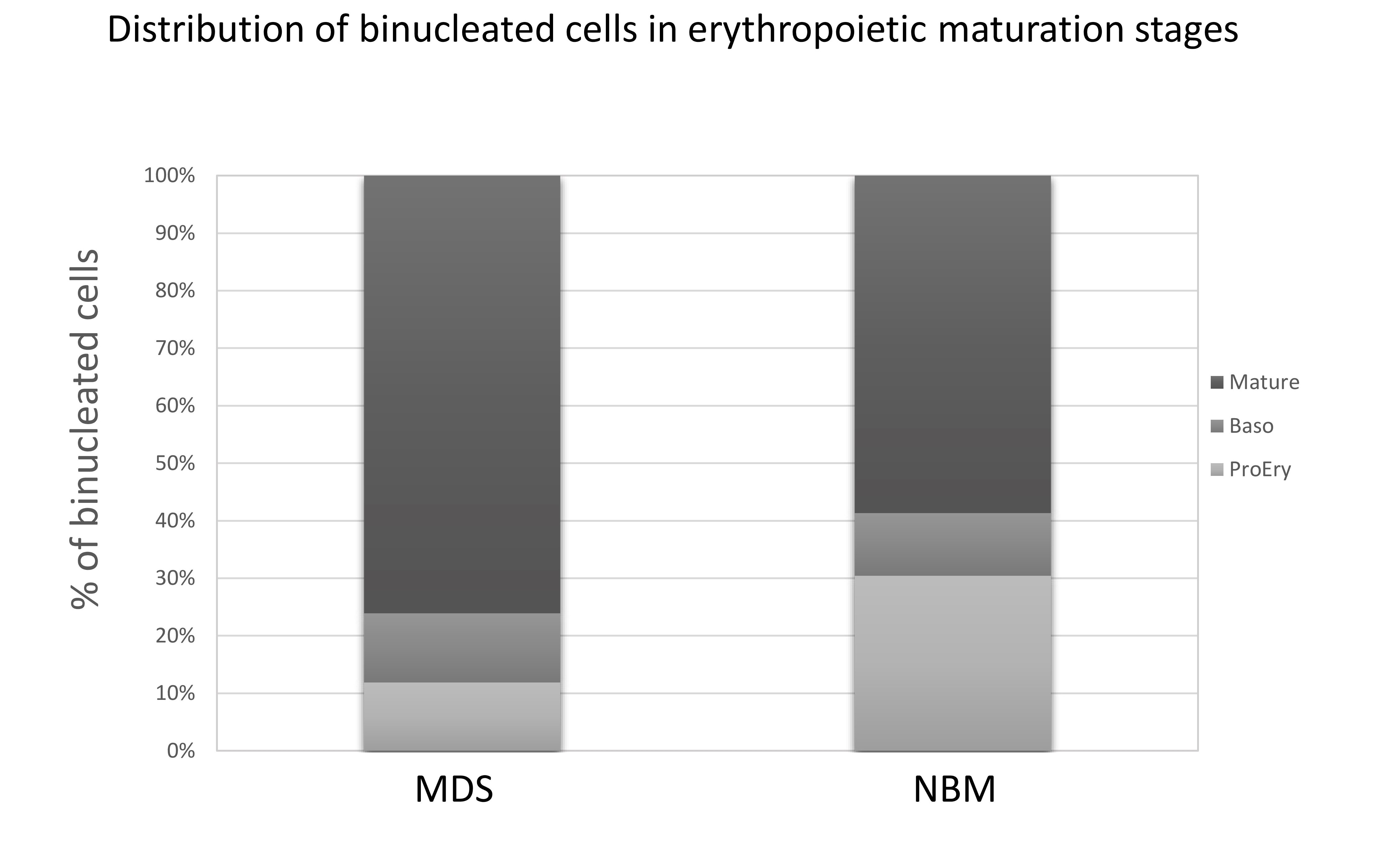

Supplement: Supplementary file 5 — Figure S5. Distribution of binucleated cells in the three maturation stages, in MDS and normal BM cases. In the MDS cases, most binucleated cells (73%) were mature. In NBM there was a similar distribution of abnormal nuclei between mature (57%) and early stages (43%). [file IJLH-47-1089-s002.jpg]

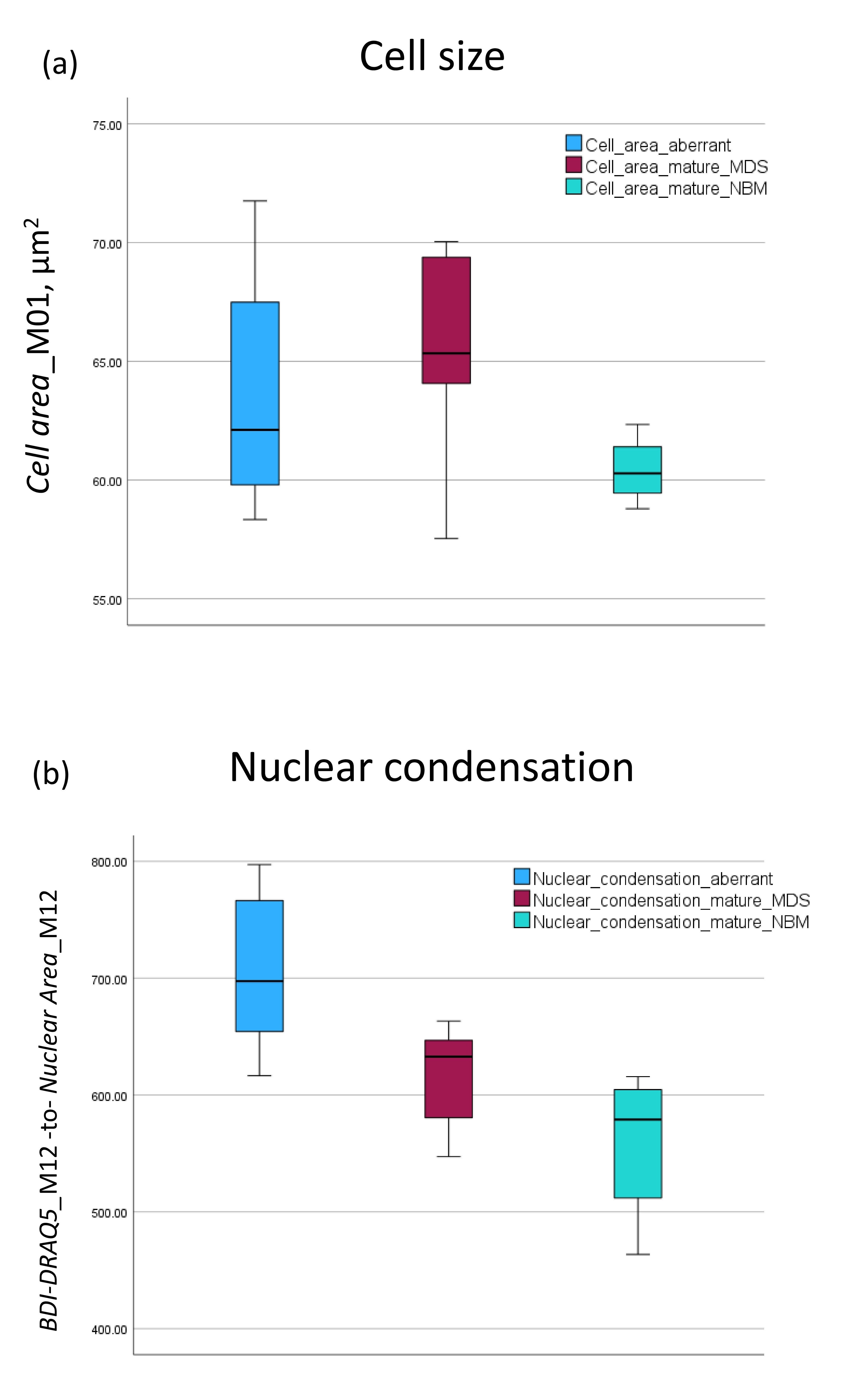

Supplement: Supplementary file 6 — Figure S6. Boxplots depicting changes in (a) cell size and (b) nuclear condensation (measured as the ratio BDI‐DRAQ5_M12 ‐to‐ Nuclear Area_M12) in the aberrant CD36−/dim ± CD71−/dim population (blue, left box), in the mature, non‐aberrant compartment of the MDS (red, middle box) and in the mature compartment of NBM (green, right box). [file IJLH-47-1089-s007.jpg]
