# Supplementary material for: Translation of the Morphological Hallmarks of Dyserythropoiesis to Objective Morphometric Parameters by Imaging Flow Cytometry
Source: Int J Lab Hematol. 2025 Jul 26;47(6):1089–98. doi: 10.1111/ijlh.14534 (PMC12597861; doi:10.1111/ijlh.14534)
Supplement: Supplementary file 7 — Data S1. [file IJLH-47-1089-s001.pdf]

## Supporting information

### Normal bone marrow samples

Ten of the normal BM specimens (NBM) were collected from patients that underwent BM investigation for lymphoma staging; one patient was referred with clinically suspected mastocytosis, but no evidence of such after completed medical investigation; one patient was investigated because of a focal lesion in the spleen, initially suspicious for lymphoma, which proved to be hemangioma (Table S1). None of the patients in the NBM group had morphological or flow cytometric evidence of BM involvement by lymphoma or other myeloid neoplasm, no previous history of treatment for malignancy and they all exhibited normal blood count and morphologically normal hematopoiesis.

### Sample staining and acquisition

250 000 cells were incubated with 2.5 µl CD71-FITC (BD Biosciences, San Jose, CA, USA), 5 µl CD105-PE, 10 µl CD117-ECD, 5 µl CD36-PB and 5 µl CD45-KO (all Beckman Coulter, Marseille, France) in the dark at room temperature (RT) for 10 minutes. After washing and resuspension by gentle vortexing, the sample was incubated with 10 µl of 0.5 mM DRAQ5 (BioStatus, Leicestershire, UK) in the dark at RT for 15 min. Compensation tubes for each antibody and for DRAQ5 were separately prepared and analysed before each daily analysis set.

Acquisition was performed on an Amnis® ImageStream®$^{X}$ Mk II Imaging flow cytometer (previously Luminex, currently Cytek® Biosciences, Fremont, Ca, USA), equipped with two cameras (set on 60x magnification) and three lasers (405 nm, 488 nm, and 642 nm). 50 000 events were collected for each sample, with a live gate set on DRAQ5 positive events. The channel distribution of the applied panel is presented in Table S2.

**Gating strategy to retrieve the optimal cells for assessment**

In order to define the focused and single cells, we started with the two-step gating strategy proposed by the IDEAS® guided analysis wizard and applied by others (Hui et al., 2018; McGrath et al., 2017; Rosenberg et al., 2021; Smirnov et al., 2015).

We started thus with the histogram of the *Gradient Root Mean Square (RMS)* of the Brightfield image (channels 1 and 9) to collect the cells with best optical focus ("focused", Figure S1a), followed by the scatter plot of the *Cell Area*_M01 vs the *Aspect Ratio*_M01 to determine the single cells ("Singles", Figure S1b). We added one extra step, using a histogram displaying the intensity of DNA DRAQ5 staining, in order to exclude the non-viable or apoptotic events (that exhibit low DRAQ5 intensity) (Figure S1c).

After these initial gating steps, reviewing of the Brightfield pictures revealed artefacts in some specimens, for example not properly centered, cropped cells, small particles included in the image and some remaining doublets (Figure S1e). These artefacts of course affected the shape and size of the cells and the derived masks and could therefore affect the morphometric calculations. In order to include centered cells only, we used the centroid features, which estimate the distance of the center of a mask from the leftmost, upper corner of the image. Different centroid features are available, and the one that gave optimal results was the *Centroid X* of the brightfield cell mask M01-erode. To further help removing cropped cells and artefacts affecting the cell shape we used the *Circularity* feature of the brightfield cell mask M01-erode. Finally, these two parameters were plotted together in a bivariate scatter plot leading to the "optimal" for assessment cell population ("Optimal", Figure S1d).

After each gating we always performed visual inspection of cells included in and excluded from the selected gates to confirm that the various populations were correctly located.

Because the default masks are quite broad, often covering pixels outside the observed cell or nuclear area (Figure 1b), we applied the erode function in the most frequently used masks, that is the Brightfield mask M01 (used to define the whole cell area) and the nuclear mask of DRAQ5, M12 (Figure 1c). This function creates tighter more representative areas and is adopted by most studies using IFC for the study of hematopoiesis (McGrath et al., 2017; Pelletier et al., 2017; Rosenberg et al., 2021). For the sake of simplicity, we have emitted the designation "erode" from the mask name, and, unless otherwise specified, the mask names M01 and M12 stand for their eroded versions.

**Gating strategy for the erythroid population**

As in our previous work (Violidaki et al., 2020), we started the retrieval of erythropoiesis by gating the $CD36^{+}/CD45^{-}$ population in the scatter plot of Intensity CD45 vs Intensity CD36 (yellow, Figure S2a). It is known that the earliest erythroid precursors exhibit some weak CD45 positivity and overlap thus with other progenitors. We added therefore the scatter plot of Intensity CD45 vs Intensity CD105, where the $CD105^{+}$ earliest erythroids can be readily gated as $CD105^{+}/CD45^{dim}$ (red, Figure S2b). The final "Erythropoiesis" population is then created with Boolean logic as $CD36^{+}/CD45^{-}$ OR $CD105^{+}/CD45^{dim}$. Erythropoiesis is further divided into three phenotypical maturation stages: $CD117^{+}/CD105^{+}$ ProEry, $CD117^{-}/CD105^{+}$ Baso and $CD117^{-}/CD105^{-}$ Mature (Figure S2c). Other cell populations (granulopoiesis, monocytes, lymphocytes) could be gated approximately based on their CD45 and CD36 characteristics (Figure S2d).

**Gating strategy for the detection of binucleated erythroblasts**

The IDEAS® software provides many features for the evaluation of shape. The features that we found useful for the evaluation of abnormal nuclear shape were *Symmetry*, *Circularity*, *Compactness* and *Lobe Count* (Table S3).

To detect binucleated erythroid progenitors, we started the retrieval by applying the *Lobe Count* feature on the eroded nuclear mask of DRAQ5, M12 ("Bilobed", Figure S3a) on all erythropoietic cells. This feature is based on the values of the features *Symmetry 2, 3* and *4* which measure the tendency of an object to have a single, a three-fold or a four-fold axis of elongation, respectively. The use of the *Lobe Count* feature resulted in various numbers of putative binucleated cells (mean 307, range 67-1095, for all 38 cases investigated). By visually inspecting these cells, we realized that most were in fact not binucleated but did exhibit abnormally shaped nuclei, including elongated or indented nuclei (Figure S3c, "Bilobed" in bottom image gallery).

In order to find real binucleates, we chose five MDS cases with an amount of presumably binucleated cells closer to the mean count of 307 to visually inspect the brightfield pictures combined with the DRAQ5 image (CH01/DRAQ5) and hand-pick the "real binucleates" (Figure S3b, Real binucleated in top image gallery). We then used this population of true binucleated erythroblasts to define which features best characterize it. To this end, we applied the Feature Finder Wizard of the IDEAS® software to compare the real binucleates with uninucleate erythropoiesis (excluding the real binucleated cells from the total of erythropoiesis with the Boolean logic "Erythropoiesis AND NOT real binucleates"), as well as with the presumably binucleated population ("Bilobed") retrieved by the *Lobe Count* feature (again, excluding the real binucleated cells with the Boolean logic). The Feature Finder Wizard ranks the features that better distinguish between two selected populations, based on the RD Mean, i.e., the

Fischer's discriminant ratio (the difference in the means divided by the sum of the standard deviations for the two populations). The top ranked shape features were *Circularity*, *Symmetry 2, Aspect Ratio Intensity* and *Compactness*, all of them applied on the eroded nuclear mask of DRAQ5 (M12).

We then combined these features in two bivariate scatter plots, *Symmetry 2* vs *Circularity* and *Compactness* vs *Aspect Ratio Intensity*, where we plotted the real binucleates that were hand-picked from the five random cases (Figure S3b). Using the minimum and maximum of the respective feature values for this real binucleated population, we created sequential detection gates in these two bivariate plots (Figure S3b). The goal was to define a population ("presumable binucleates", Figure S3c) containing the real binucleates contaminated as less as possible with non-binucleates. Therefore, in the next step we applied and sequentially gated in these plots the "bilobated" population as retrieved by the *Lobe Count* feature (Figure S3c). Even when we used the total erythropoiesis as a starting population, skipping the *Lobe Count* feature, we ended up in similar numbers of the final presumably binucleated population (data not shown).

Overlapping with non-binucleated cells was inevitable in the final detection gate (named Presumable binucleates, Figure S3c) and as expected, even these cells showed abnormal nuclear shape, being cells exhibiting feature values (low *Circularity*, low *Aspect Ratio*, low *Compactness*, high *Symmetry 2*) deviating from the "normal".

The aim of our approach remained therefore to obtain the smallest number of objects with abnormally shaped nuclei that would include the real binucleated population. Thus, by limiting the number of cells included in the detection gates, we could facilitate the visual inspection and tracing of the real binucleates by using the combined brightfield/DRAQ5 images.

**References**


Haralick, R. M., Dinstein, I., & Shanmugam, K. (1973). Textural Features for Image Classification. *IEEE Transactions on Systems, Man and Cybernetics*, *SMC-3*(6). https://doi.org/10.1109/TSMC.1973.4309314

Hui, H., Fuller, K. A., Chuah, H., Liang, J., Sidiqi, H., Radeski, D., & Erber, W. N. (2018). Imaging flow cytometry to assess chromosomal abnormalities in chronic lymphocytic leukaemia. *Methods*, *134–135*, 32–40. https://doi.org/10.1016/j.ymeth.2017.11.003

McGrath, K. E., Catherman, S. C., & Palis, J. (2017). Delineating stages of erythropoiesis using imaging flow cytometry. *Methods*, *112*, 68–74. https://doi.org/10.1016/j.ymeth.2016.08.012

Pelletier, M. G. H., Szymczak, K., Barbeau, A. M., Prata, G. N., O'Fallon, K. S., & Gaines, P. (2017). Characterization of neutrophils and macrophages from ex vivo-cultured murine bone marrow for morphologic maturation and functional responses by imaging flow cytometry. *Methods*, *112*, 124–146. https://doi.org/10.1016/j.ymeth.2016.09.005

Rosenberg, C. A., Bill, M., Rodrigues, M. A., Hauerslev, M., Kerndrup, G. B., Hokland, P., & Ludvigsen, M. (2021). Exploring dyserythropoiesis in patients with myelodysplastic syndrome by imaging flow cytometry and machine-learning assisted morphometrics. *Cytometry Part B - Clinical Cytometry*, *100*(5), 554–567. https://doi.org/10.1002/cyto.b.21975

Smirnov, A., Solga, M. D., Lannigan, J., & Criss, A. K. (2015). An improved method for differentiating cell-bound from internalized particles by imaging flow cytometry. *Journal of Immunological Methods*, *423*, 60–69. https://doi.org/10.1016/j.jim.2015.04.028

Violidaki, D., Axler, O., Jafari, K., Bild, F., Nilsson, L., Mazur, J., Ehinger, M., & Porwit, A. (2020). Analysis of erythroid maturation in the nonlysed bone marrow with help of radar plots facilitates detection of flow cytometric aberrations in myelodysplastic syndromes. *Cytometry Part B - Clinical Cytometry*, *98*(5), 399–411. https://doi.org/10.1002/cyto.b.21931

**Table S1. Clinical characteristics and diagnoses or reasons for bone marrow investigation.**

| **Normal controls NBM (n = 12)** | |
|---|---|
| *Age, years, mean (range)* | 63 (42-78) |
| *Sex (M: F)* | 6:6 |
| *Hb, g/L, mean (range)* | 140 (123-154) |
| *WBC, $x10^9$/L, mean (range)* | 6.7 (4.5-10.5) |
| *Platelets, $x10^9$/L, mean (range)* | 232 (152-366) |
| Reasons for investigation of the BM | |
| *Staging of lymphoma* | 10 (84%) |
| *Anaphylaxis investigated for mastocytosis* | 1 (8%) |
| *Focal spleen lesion investigated for lymphoproliferative disease* | 1 (8%) |
| **MDS and MDS/MPN patients (n = 26)** | |
| *Age, years, mean (range)* | 74 (59-91) |
| *Sex (M: F)* | 19:7 |
| *Hb, g/L, mean (range)* | 102 (84-123) |
| *WBC, $x10^9$/L, mean (range)* | 6.0 (1.7-23.5) |
| *Platelets, $x10^9$/L, mean (range)* | 175 (38-637) |
| Diagnostic groups of MDS & MDS/MPN patients according to WHO 4th Edition 2017[a] | |
| *MDS - MLD* | 6 (23%) |
| *MDS - RS - MLD* | 8 (31%) |
| *MDS - EB1* | 3 (11.5%) |
| *MDS - EB2* | 3 (11.5%) |
| *MDS with isolated del(5q)* | 1 (4%) |
| *MDS/MPN* [b] | 5 (19%) |

Abbreviations: MDS-EB: MDS with excess blasts, MDS-MLD: MDS with multilineage dysplasia, MDS/MPN: Myelodysplastic/Myeloproliferative neoplasms, MDS-RS-MLD: MDS with ring sideroblasts and multilineage dysplasia

[a] The cases were received and diagnosed between 2018-2020.

[b] The 5 MDS/MPN cases were 2 MDS/MPN with ring sideroblasts and thrombocytosis (MDS/MPN-RS-T), 2 chronic myelomonocytic leukemias (CMML) and one MDS/MPN-unclassifiable (MDS/MPN-U)

**Table S2. Channel distribution of the brightfield images and applied antibodies/dyes.**

| FL1 | FL2<br>FITC | FL3<br>PE | FL4<br>ECD | FL5 | FL6 | FL7<br>PB | FL8<br>KO | FL9 | FL10 | FL11 | FL12 |
|---|---|---|---|---|---|---|---|---|---|---|---|
| **BF*** | **CD71** | **CD105** | **CD117** | **-** | **SCC#** | **CD36** | **CD45** | **BF** | **-** | **-** | **DRAQ5** |

*BF: Brightfield

#SCC: Side Scatter

**Table S3. List of IDEAS® features used in the study.**

| Feature name | Description |
|---|---|
| *Area* | A size feature measuring the area of a mask in square microns. It is calculated by the number of pixels included in a mask, where 1 pixel = $0.25 \mu m^2$ |
| *Aspect Ratio* | A shape feature measuring how round or oblong a mask is. It is calculated as the ratio of the Minor Axis divided by the Major Axis. |
| *Aspect Ratio Intensity* | A shape feature measuring how round or oblong an intensity weighted mask is. It is calculated as the ratio of the intensity weighted Minor Axis divided by the intensity weighted Major Axis. |
| *Bright Detail Intensity* | A texture feature measuring the intensity of localized bright spots within the masked area. |
| *Centroid X* | A location feature measuring the number of pixels in the horizontal (x) axis form the upper left corner to the center of the mask. |
| *Circularity* | A shape feature measuring how much a mask deviates from a circle. It is calculated as the average distance of the mask boundary from its center, divided by the variation of this distance. |
| *Compactness* | A shape feature measuring how well an object is packed together. |
| *Gradient RMS* | A texture feature measuring the sharpness quality and thus the focus of an image. Its calculation is based on the detection of large changes of pixel values in the image, using the average gradient of a pixel, normalized for variations in intensity levels. |
| *H Contrast* | A texture feature measuring the intensity variation in an area. |
| *H Correlation* | A texture feature measuring how similar adjacent pixels are; it is the opposite of contrast. |
| *H Energy* | A texture feature measuring intensity concentration in an area. |
| *H Entropy* | A texture feature measuring randomness of intensity concentration in the cell. Images with distinct areas of intensity concentration are less random and thus have low entropy. Entropy is the opposite to energy. |
| *H Homogeneity* | A texture feature measuring how close pixel values are. Images with high homogeneity would look very uniform and lack texture. |
| *H Variance* | A texture feature measuring the spread of pixel values within the granularity. Images with high variance will have very dark and very bright spots. |

| | |
|---|---|
| *Major Axis* | A size feature measuring the longest dimension of an ellipse of best fit for a specific mask. |
| *Minor Axis* | A size feature measuring the narrowest dimension of the ellipse of best fit for a specific mask. |
| *Lobe Count* | A shape feature measuring the lobes of a mask. It is calculated based on the maxima of the weighted Symmetry features. |
| *Symmetry 2, 3, 4* | A shape feature measuring the tendency of a mask to have a single or many-fold axis of elongation. Single axis corresponds to 2 lobes, three-fold axis to 3 lobes and four-fold axis to 4 lobes. |

**Table S4. Percentages (% of erythropoiesis) of cells with abnormal nuclei and real binucleates in MDS and NBM.**

| | Cells with abnormal nuclei (mean / median) | | Real binucleates (mean / median) | |
|---|---|---|---|---|
| NBM | 9.1 | 9.2 | 0.4 | 0.4 |
| MDS | 11.5 | 10.9 | 0.9 | 0.9 |
| *p*-value NBM vs MDS | .017 | | <.001 | |

**Table S5. Values of *Cell Area*_M01 (“cell size”) in NBM and MDS in µm² (mean ± SD)**

| | Total erythropoiesis | ProEry | Baso | Mature |
|---|---|---|---|---|
| **NBM** | 65.6 ± 1.8 | 98.5 ± 5.4 | 83.9 ± 3.7 | 62.2 ± 1.6 |
| **MDS** | 70.7 ± 4.6 | 107.5 ± 6.8 | 91.4 ± 5.4 | 67.6 ± 4.6 |
| *p*-value NBM vs MDS | < .001 | < .001 | < .001 | < .001 |
